# Preoperative embolization and lung-sparing robotic surgery in intrathoracic Castleman’s disease: case report

**DOI:** 10.1590/1677-5449.202501672

**Published:** 2026-03-23

**Authors:** Felipe Furtado Leite, Ricardo Sales dos Santos, Priscila Mina Falsarella, Kauê Polizel Souza, Breno Boueri Affonso, Leonardo Guedes Moreira Valle, Mario Cláudio Ghefter, Felipe Nasser

**Affiliations:** 1 Universidade Federal da Bahia – UFBA, Salvador, BA, Brasil.; 2 Faculdade de Ciências Médicas da Santa Casa de São Paulo, São Paulo, SP, Brasil.; 3 Instituto Israelita de Ensino e Pesquisa Albert Einstein, São Paulo, SP, Brasil.; 4 Faculdade de Medicina do ABC – FMABC, Santo André, SP, Brasil.; 5 Universidade de Sao Paulo – USP, São Paulo, SP, Brasil.

**Keywords:** Castleman disease, robotic surgical procedures, therapeutic embolization, mediastinum, minimally invasive surgery, case reports, doença de Castleman, cirurgia robótica, embolização terapêutica, mediastino, cirurgia minimamente invasiva, relato de caso

## Abstract

Castleman’s disease is a rare lymphoproliferative disorder that may present as unicentric or multicentric, with differing therapeutic implications. We report the case of a 23-year-old woman with unicentric Castleman’s disease in the mediastinum. Following unsuccessful systemic therapy with corticosteroids and monoclonal antibodies, she underwent successful preoperative embolization using Embosphere® microspheres and subsequent robotic-assisted resection. This approach minimized blood loss (<100 mL) and facilitated safe dissection in a surgically challenging region. The patient had an uneventful postoperative course and was discharged on postoperative day seven. This case highlights the benefits of combining embolization with robotic surgery in managing hypervascular mediastinal lesions, offering a minimally invasive and safe alternative in selected patients.

## Resumo

A doença de Castleman é uma doença linfoproliferativa rara que pode se apresentar como unicêntrica ou multicêntrica, com diferentes implicações terapêuticas. Relatamos o caso de uma mulher de 23 anos de idade com doença de Castleman unicêntrica no mediastino. Após terapia sistêmica com corticosteroides e anticorpos monoclonais sem sucesso, a paciente foi submetida a embolização pré-operatória bem-sucedida, com microesferas Embosphere® e subsequente ressecção assistida por robô. Essa abordagem minimizou a perda sanguínea (<100 mL) e facilitou a dissecção segura em uma região cirurgicamente desafiadora. A paciente teve um pós-operatório sem intercorrências e recebeu alta no sétimo dia de pós-operatório. Este caso destaca os benefícios da combinação de embolização com cirurgia robótica no manejo de lesões mediastinais hipervasculares, oferecendo uma alternativa minimamente invasiva e segura em pacientes selecionados.

## INTRODUCTION

Castleman’s disease (CD), described in 1954 by Dr. Benjamin Castleman, encompasses a spectrum of rare lymphoproliferative disorders that can cause life-threatening complications, whether in their unicentric or multicentric form.^1^ The incidence of unicentric CD in the USA is approximately 16 cases per million per year and incidence of the multicentric form is approximately 5 cases per million per year.^2^ The condition’s effects include systemic symptoms secondary to a compressive condition and possible pulmonary complications, for example, among others such as autoimmune hemolytic anemia.^1,3^

Definitive treatment is complete surgical excision for unicentric disease. However, surgical resection can be challenging in cases of hypervascular lesions and may lead to excessive blood loss, major dissections, or even inadvertent injury to other organs.^3-5^

In this context, perioperative embolization emerges as an extremely valuable tool after failure of treatment with corticosteroids, immunobiologicals, and other pharmacochemical approaches, corroborating surgical indications in these more challenging cases.^5^

This report describes the case of a patient who underwent preoperative embolization followed by robotic excision of CD in the mediastinal compartment.

This study was approved by the institutional review board and informed consent was obtained. The Research Ethics Committee approved this study (decision number 7.187.370; CAAE: 83846524.8.0000.0071).

## CASE REPORT

A 23-year-old female patient sought medical attention due to an incidental finding of a possible chest mass on a chest X-ray. The physical examination was normal during the initial evaluation.

The patient’s medical history included asthma, hypothyroidism with Graves’ disease on iodine therapy, and obesity, with no family history of lymphomas/leukemia.

**Figure 1:**
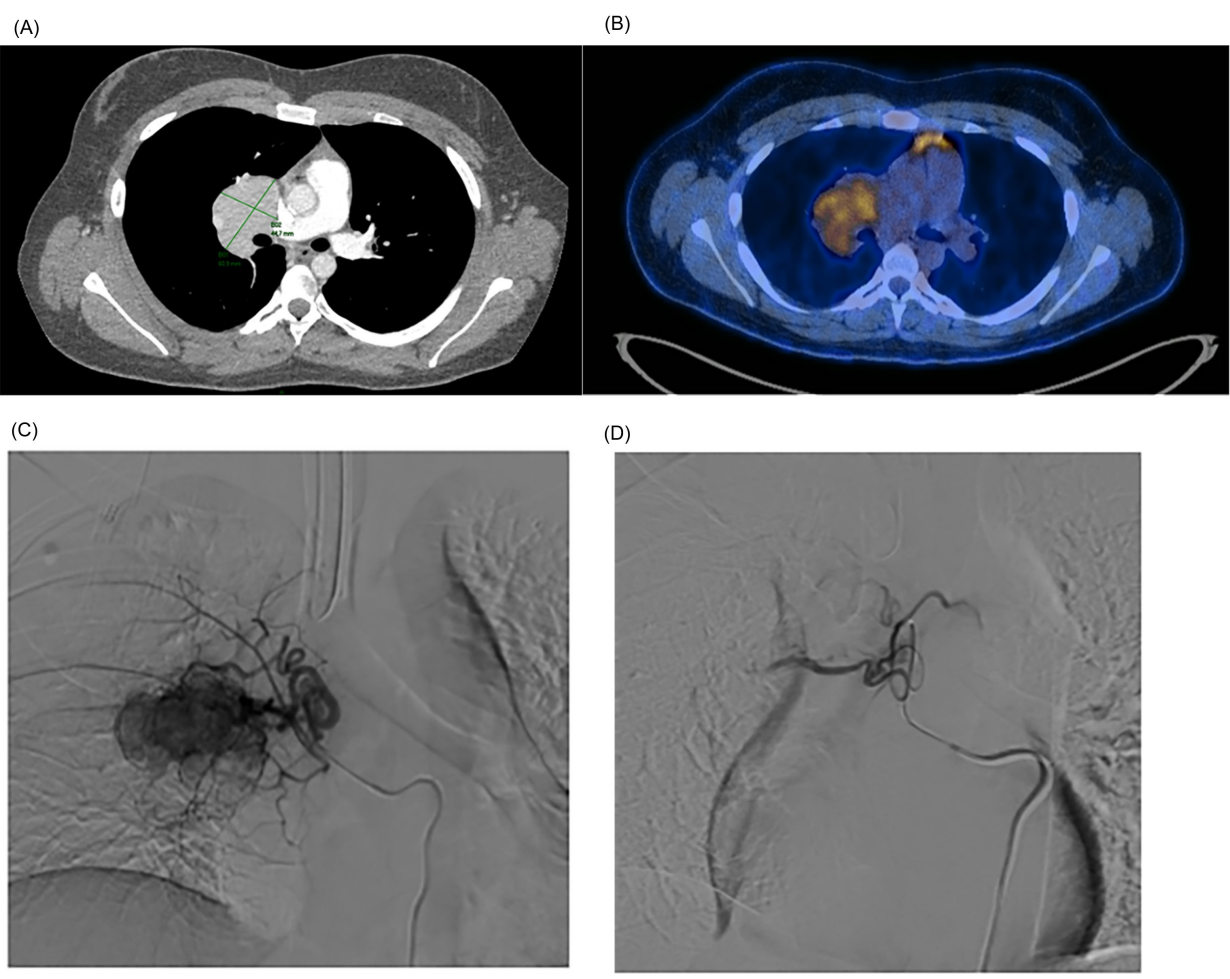
(A) Contrast-enhanced CT with axial section, at the level of the bifurcation of the pulmonary artery and the main bronchi, showing the mass in the right pulmonary hilum, measuring 6.1 × 4.5 cm; (B) PET-CT axial section with fusion of metabolic images with fluorodeoxyglucose-18F (FDG) showing radiopharmaceutical uptake with SUV of up to 4.5; (C) Selective catheterization of the right bronchial artery ostium with a 5 Fr Mikaelson catheter; (D) Angiographic control after embolization with Embosphere® Merit 300-500 microns microspheres, showing an outcome with contrast stasis in the feeding trunk branch.

After the initial consultation, a computed tomography (CT) scan of the chest was ordered, which showed a mass in the right pulmonary hilum, with homogeneous contrast enhancement, measuring 6.1 x 4.5 cm at the largest axial axes, involving the regional bronchovascular structures, and without significant obstructive effect. A positron emission tomography (PET/CT) study was performed, which demonstrated radiopharmaceutical uptake with standardized uptake value (SUV) of up to 4.5 in the mass (Figures 1A and 1B) and lymphoproliferative diseases were included among the differential diagnoses.

She underwent a transthoracic biopsy guided by CT, the final histopathological analysis of which showed no evidence of aggressive lymphoproliferative disease.

The patient was discharged from hospital in December 2021, with indication for monitoring/control of the lesion by imaging, as the mediastinal finding was of reactive origin, ruling out the possibility of hematologic lineage neoplasia.

In 2022, she underwent thoracotomy and biopsy in another hospital unit, evolving with an episode of significant intraoperative bleeding with a chest tube remaining for several days. The anatomopathological result of the external service indicated CD. Treatment with siltuximab (2 cycles), corticosteroids, and rituximab (December 2022 and February 2023) was then chosen. Control with PET-CT after treatment showed persistent hyperuptake of the radiopharmaceutical, with a slight increase in the degree of metabolic activity in the mediastinal mass. Due to the complexity of the case and high risk of pneumonectomy, the surgical option was not considered.

In April 2024, after 12 cycles of siltuximab and 8 doses of rituximab, a new contrast-enhanced CT showed no significant radiological changes and it was decided to refer the patient for a new evaluation with the thoracic surgery team. After multidisciplinary discussion of the case together with the interventional radiology team, a new surgical approach with preoperative embolization was chosen.

**Figure 2:**
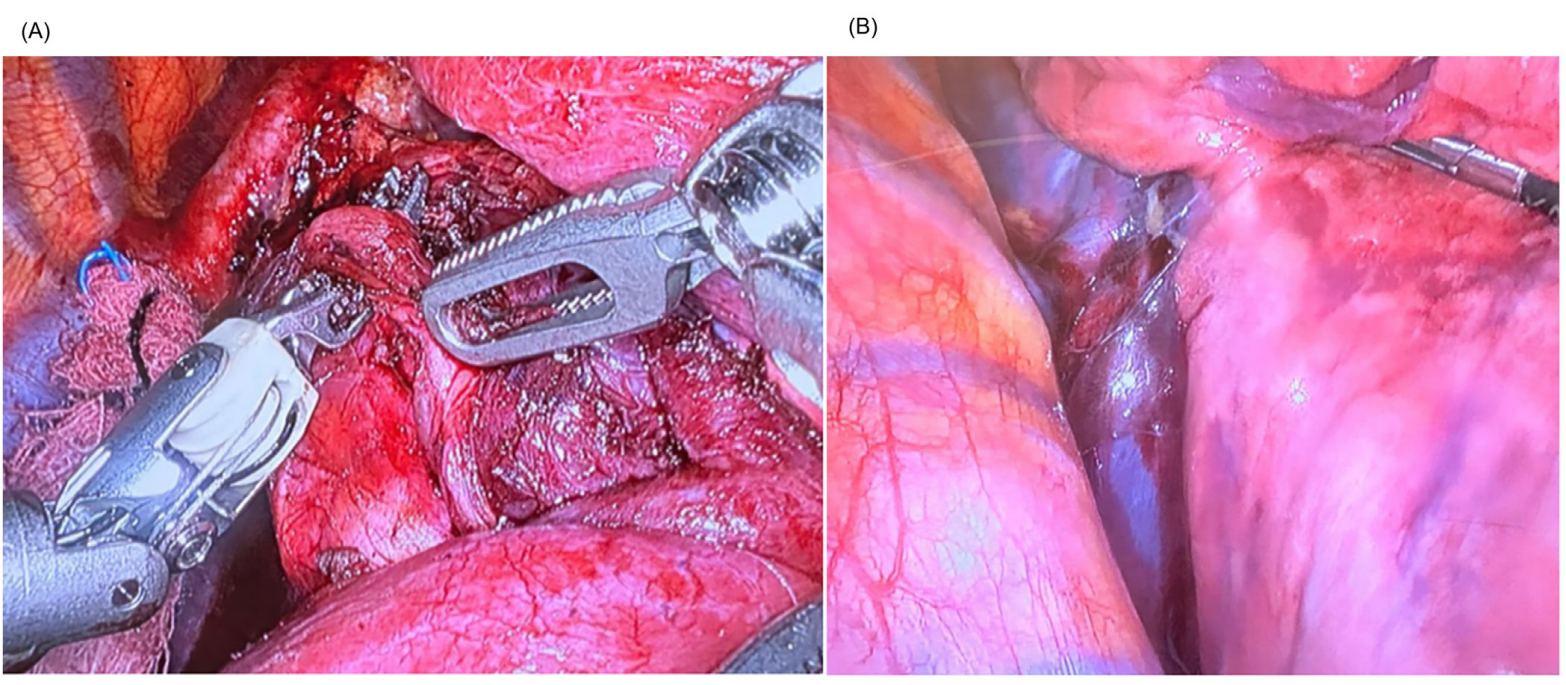
(A) Intraoperative image of hematoma after embolization of the hilar lesion; (B) Intraoperative image of the moment of lesion resection, highlighting the caliber of the previously embolized bronchial artery.

**Figure 3:**
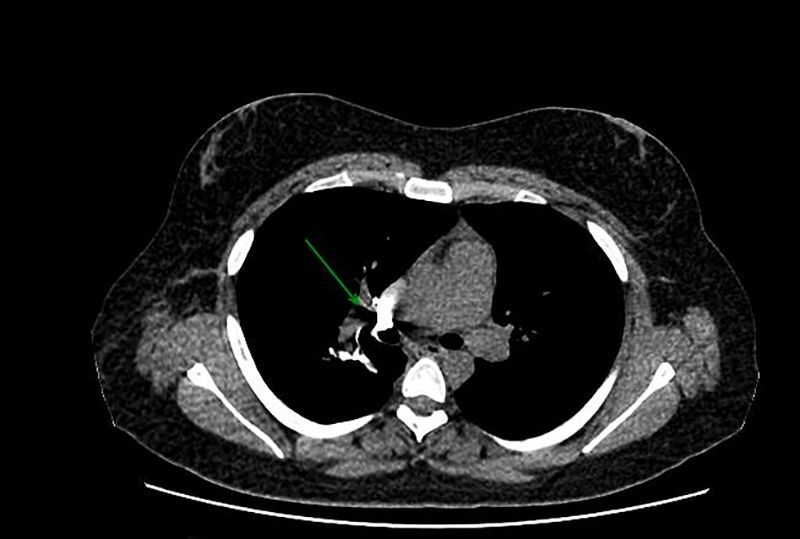
Non-contrast chest CT scan showing signs of surgical manipulation in the right pulmonary hilum, with clips and peri-hilar metallic suture, with slight prominence of the hilar region.

The patient underwent preoperative embolization of the mass with 300 to 500 micron Embosphere® Merit microspheres (Figures 1C and 1D), followed by excision of the mediastinal lesion by robotic-assisted video-assisted thoracic surgery (RVATS), with blood loss of less than 100 ml (Figure 2). The patient showed good clinical evolution and was discharged after seven days in hospital. The patient remains in outpatient follow-up. She underwent PET/CT at 3 months and a non-contrast chest CT scan at 6 months, showing no signs of tumor recurrence (Figure 3).

## DISCUSSION

Unicentric CD is usually benign, with good prognosis after complete surgical removal. It is crucial to differentiate between the unicentric and multicentric forms of the disease, as the multicentric form may be more aggressive and requires personalized management.^3,5^

Surgical manipulation of lesions located close to the pulmonary hilum presents a complex challenge. Use of the RVATS system for such lesions allows better visualization and dexterity during the procedure performed in a minimally invasive manner; however, hypervascularized lesions constitute an additional risk, since mobilization can cause major bleeding, even if there is no invasion of the pulmonary vasculature. From an anatomical point of view, hilar neoplastic lesions are irrigated by bronchial arterial branches, which derive directly from the aorta and are therefore at high pressure.^5-7^

Robotic surgery has proven to be a fundamental tool in the management of complex thoracic injuries. The precision offered by robotic surgery, with the possibility of performing detailed movements in difficult-to-access areas, facilitates careful dissection of critical vascular and lymphatic structures.^7-9^ This approach proved especially useful in the reported case, where tissue preservation was essential to minimize the risk of inadvertent injury to adjacent organs.

Hypervascular lesions benefit significantly from the combination of preoperative embolization and robotic surgery. This integrated management approach results in reduced intraoperative complications, such as excessive blood loss, while improving control of hemostasis during lesion resection.^8,9^ In the reported case, preoperative embolization followed by RVATS mediastinal excision resulted in minimal blood loss and hospital discharge after seven days, reflecting the benefits of this combined approach.

Furthermore, robotic surgery allows for faster recovery compared to traditional open approaches. In the context of injuries that would normally require open thoracotomy from the outset, the minimally invasive approach afforded by robotics offers significant advantages, allowing for preservation of adjacent organs and safer navigation in complex surgical fields.^9,10^

Currently, the specialty of vascular surgery has evolved in the use of minimally invasive techniques, known as endovascular techniques. This article describes an endovascular technique aimed to reduce the risk of bleeding during the surgical procedure. It also offers guidance for vascular surgeons regarding the anatomical aspects associated with this approach and the therapy employed.

In conclusion, although this is a single case report, with consequent limitation on the extrapolation of results, the preoperative embolization technique allows navigation of more complex surgical fields. New studies addressing this topic need to be carried out and we emphasize that the operator’s skill and familiarity with the embolization technique is a crucial factor for success.

## Data Availability

All data generated or analyzed are included in this article and/or in the supplemental material.
